# Standing HA phenotypic breadth shapes H5N1 cross-host potential

**DOI:** 10.21203/rs.3.rs-10349790/v1

**Published:** 2026-07-21

**Authors:** M H M Mubassir, Sachin Subedi, Tanin Rajamand, Mohamed Bakheet, Ludy Registre Carmola, Sihua Peng, Rajan Kandel, Guppy Stott, Robert J. Woods, S. Mark Tompkins, Gerardus Josephus Boons, Justin Bahl

**Affiliations:** 1 Institute of Bioinformatics, University of Georgia, Athens, GA, United States.; 2 Center for Ecology of Infectious Diseases, University of Georgia, Athens, GA, United States.; 3 Department of Infectious Diseases, College of Veterinary Medicine, University of Georgia, Athens, GA, United States.; 4 Department of Epidemiology and Biostatistics, College of Public Health, University of Georgia, Athens, GA, United States.; 5 Complex Carbohydrate Research Center, University of Georgia, Athens, GA, United States.; 6 Department of Chemistry, University of Georgia, Athens, GA, United States; 7 Department of Biochemistry and Molecular Biology, University of Georgia, Athens, GA, USA; 8 Center for Vaccines and Immunology, University of Georgia, Athens, GA, United States

## Abstract

Linking genetic variation to functional phenotype remains a major barrier to assessing the cross-host potential of emerging viruses. Here, we reconstruct the evolution of predicted hemagglutinin (HA) phenotypic traits across ~13,000 highly pathogenic avian influenza A H5N1 clade 2.3.4.4b viruses circulating in North America. The wide geographic spread of avian influenza within North America triggered a wave of broad HA phenotypic diversity that was later refined by the selective sweeps in avian hosts. Following establishment in dairy cattle, however, viral populations exhibited renewed phenotypic diversification in HA, including increased permissiveness for α2,6-linked sialoside engagement despite high conservation of the receptor-binding domain. These patterns indicate that cattle-associated HA phenotypes can draw from standing predicted HA phenotypic breadth within circulating viral populations while continuing to diversify during cattle-associated circulation, rather than following only a simple stepwise adaptive path through canonical receptor-binding substitutions. By linking viral sequence variation to predicted protein properties across naturally evolving populations, this framework provides a scalable strategy for prioritizing H5N1 variants with cross-host-relevant features for targeted surveillance and experimental follow-up.

## Introduction

Highly pathogenic avian influenza A H5N1 clade 2.3.4.4b is undergoing a rapid host expansion across North America, establishing sustained transmission in mammals while continuing to diversify in birds [1–4]. The dairy epizootic is especially important because it represents prolonged replication in a mammalian host with dense contact networks, new transmission routes, and increased occupationally associated human infections [3, 5–7]. Despite extensive genomic surveillance, assessment of cross-host potential remains limited by our ability to translate naturally occurring sequence variation into quantitative HA phenotypes and to track how those phenotypes evolve across viral populations.

The host range of influenza A is strongly influenced by the viral surface protein hemagglutinin (HA), whose receptor binding specificity, stability, and fusion properties jointly determine infection and transmission [8–10]. However, HA is only one component of the host-range barrier, and receptor engagement alone does not determine infection, replication, transmission, or pandemic risk. Yet most mechanistic studies focus on individual “switch” mutations, interrogating only a narrow region of sequence space and providing limited insight into how naturally circulating viruses traverse multidimensional phenotype space over time [11–13]. This limitation is consequential because adaptation may not require classical receptor-binding substitutions, and recent comparative phylogenetic analyses suggest that extensive pre-zoonotic adaptive optimization is often not detectable prior to emergence [8, 14–19]. Viruses with latent functional breadth can establish in novel hosts and diversify before recognizable adaptive mutations arise, implying that cross-species emergence may depend on population-level phenotypic breadth, rather than the appearance of a single high-risk genotype [20, 21]. This broad principle is established, but it remains difficult to measure how standing sequence variation translates into predicted HA phenotypes within a densely sampled, naturally evolving viral population [22–24]. These observations highlight the need for lineage-resolved approaches that connect naturally occurring genetic variation to quantitative HA phenotypes.

Here, we address this gap by constructing a sequence–structure–function landscape for U.S. clade 2.3.4.4b hemagglutinins, linking naturally sampled sequence diversity to a broad panel of predicted HA traits that can be mapped onto a phylogeny to capture the temporal phenotypic variation associated with interspecies transmission and geographic expansion. Using ~13,000 HA sequences (2,195 unique amino acid variants), we integrate sequence-derived features, including net charge, hydrophobicity, and secondary structure variation, with structure-based estimates of thermodynamic stability, and functional estimates of lineage-specific receptor-binding dynamics, traits expected to influence HA receptor usage, fusion behavior, and thus to host range and cross-species transmission.

Embedding phenotypic properties directly within evolutionary history reveals where functional diversity concentrates along outbreak lineages and provides a mechanistic framework for prioritizing HA variants with cross-host-relevant features for targeted surveillance and experimental follow-up as H5N1 continues to diversify across birds, cattle, and other mammals. This approach reframes surveillance from tracking individual mutations alone toward quantifying the functional potential of circulating viral populations.

## Results

### Population expansion generates phenotypic diversity that is rapidly pruned by selection

**Figure 1.**
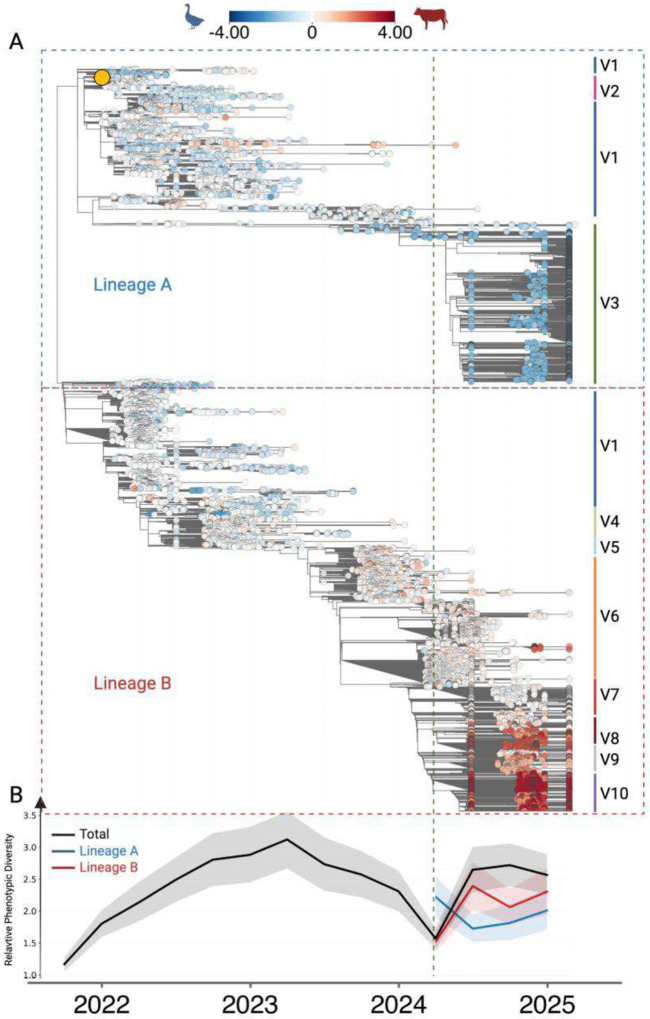
Expansion and selective sweeps structure phenotypic diversity in the U.S. H5N1 population. (A) Phylogenetic reconstruction of ~13,000 U.S. H5N1 HA sequences (2021–2025). The 2021 reference virus (V1) is indicated by a yellow circle. Tip colors represent the mean phenotypic shift relative to the reference virus, projected along an avian-to-cattle phenotypic axis defined by the avian-associated variant V3 (blue) and the cattle-associated variant V10 (red). Intermediate colors indicate variants with phenotypes closer to the reference state (white). Frequently circulating variants (V1–V10) are indicated at right. (B) Trajectories of population-level phenotypic diversity. Phenotypic diversity increases during geographic expansion and peaks in 2023 before collapsing in a selective sweep (green dashed line). After this bottleneck, diversity is shown separately for the avian-associated lineage (blue) and the cattle-associated lineage (red). Shading indicates 95% confidence intervals.

Following its introduction into North America in late 2021, H5N1 clade 2.3.4.4b rapidly diversified across the United States, providing an opportunity to examine how genetic expansion translates into population-level predicted HA phenotypic diversity. To quantify how this diversification translated into functional variation, we mapped phenotypes (sequence-derived biophysical metrics, AlphaFold3-based structural diversity, and Rosetta/MD-derived stability and receptor-binding estimates) onto the HA phylogeny using an overall phenotype score summarizing diversity across core traits. This analysis included 2,195 unique HA amino acid variants, with the ten most frequently circulating variants (V1–V10) highlighted as reference points (Fig. 1, Supplementary Fig.1A). Because these variant classes are defined from HA amino-acid sequences, they should be interpreted as HA variant classes rather than whole-genome genotype designations. In the time-scaled phylogeny, tip colors represent the mean phenotypic shift relative to the 2021 reference virus (V1), anchored between the avian-associated phenotype represented by V3 (blue) and the cattle-associated phenotype represented by variants V10 (red) (Fig. 1A). Lineage A and Lineage B were defined from the HA phylogeny and predicted HA phenotype trajectories; these labels are used here as HA-centered descriptors and are not intended to replace whole-genome genotype classifications such as B3.13 or D1.1. To clarify how these HA lineages relate to host sampling, we summarized host composition across the full tree: Lineage A was predominantly avian, whereas Lineage B contained both avian viruses and most domestic-mammal/cattle-associated detections. Between 2022 and 2024, clade 2.3.4.4b expanded geographically in avian hosts, producing a wide range of predicted HA phenotypic states relative to the reference virus (V1; A/AmericanWigeon/SouthCarolina/22-006487-001/2021) (Fig. 1A, Supplementary Figs.1B–1D). Population-level phenotypic diversity increased during expansion and peaked in early 2023 (Fig. 1B), indicating extensive exploration of phenotype space. Diversity then collapsed sharply in early 2024, consistent with a selective sweep that pruned most circulating variants. After this bottleneck, the population diverged into two phenotypic trajectories: an avian-associated lineage dominated by variant V3 (Lineage A) and a second lineage comprising multiple dominant variants (V6–V10) that progressively shifted toward predicted HA profiles enriched among domestic-mammal/cattle-associated detections (Lineage B) (Fig. 1A, Supplementary Tables 1, 2). Together, these patterns indicate that geographic expansion generates extensive phenotypic diversity that is subsequently pruned by selection, leaving a small subset of variants to persist as avian and cattle-associated circulation became increasingly separated.

### Cattle-associated HA variants pre-exist within circulating avian diversity

**Figure 2.**
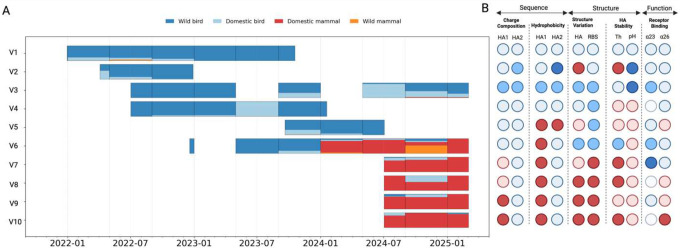
Cattle-associated HA variants circulate in birds prior to spillover. (A) Circulation timelines of the ten most frequently observed HA amino-acid variants (V1–V10). Colored bars indicate the host composition of sampled viruses within each time bin. Several variants that later dominated cattle-associated transmission first circulated in avian hosts before being detected in dairy cattle (B) Phenotypic landscape of the ten variants across sequence-, structure-, and function-based traits. Circles represent phenotype values relative to lineage reference distributions. Colors indicate lineage affinity, with blue shades reflecting avian-like phenotypes and red shades reflecting cattle-associated phenotypes. Variants that later expanded in mammals show coordinated shifts across multiple traits, including hydrophobicity, structural variation, stability, and receptor-binding phenotypes.

Following the phenotypic bottleneck identified in Fig. 1, a cattle-associated clade emerged within lineage B beginning in 2024. The V6 variant (A/dairycow/Texas/24_009775-001/2024), had already circulated in avian hosts as early as 2023 (Fig. 2A), prior to detection in cattle. Public whole-genome surveillance identifies the established U.S. dairy-cattle outbreak before early 2025 primarily with genotype B3.13, whereas genotype D1.1 was later detected in dairy cattle as a separate wild-bird-associated spillover [25]. Because our dataset and variant classes are HA-centered, we use these genotype labels only as contextual information and do not infer whole-genome genotype from HA alone. To ask whether this signal would have been visible near the start of the dairy outbreak, we repeated the variant-summary analysis using time-truncated datasets ending in March 2024. By March 1, 2024, the V6 HA background was already represented by 174 sequences from wild birds, domestic birds, and wild mammals; by March 31, it had expanded to 357 sequences, including 146 domestic-mammal/cattle-associated detections. Thus, the early surveillance signal in this dataset was the presence of the V6 HA background in avian and wild-mammal samples before rapid dairy/cattle enrichment, whereas V7–V10 represent later diversification during cattle-associated circulation.

Mapping HA phenotypes onto the phylogeny revealed that avian variants circulating between 2022 and early 2024 spanned a broad multidimensional phenotype space relative to the 2021 reference virus (V1) (Fig. 2B, Supplementary Figs.2–8), indicating that some HA backgrounds later observed in cattle-associated viruses were already present within circulating avian diversity. Following the early-2024 phenotypic bottleneck, the expanding cattle-associated lineage exhibited progressively stronger shifts in the predicted HA features enriched among domestic-mammal/cattle-associated sequences. Early cattle variants such as V6 remained largely avian-like across many traits remaining closer to lineage A, whereas later cattle-dominant variants (V8–V10) showed coordinated shifts across sequence, structural, stability, and receptor-binding phenotypes (Fig. 2B, Supplementary Figs.2–8). Together, these patterns indicate that the HA background later enriched in dairy/cattle was already present in avian diversity, followed by additional HA phenotypic change during cattle-associated circulation.

This pattern raises a mechanistic question. Many of the phenotypic changes associated with cattle transmission occur in the absence of previously identified receptor-binding substitutions, suggesting that differences in receptor engagement may arise from distributed changes around the receptor-binding site rather than from a single canonical receptor-switch substitution. These results indicate that cattle-associated HA phenotypes can build on diversity already circulating in avian viruses while continuing to evolve during cattle-associated transmission.

### Modelled receptor-binding-site geometry distinguishes avian and cattle-associated HA variants

These observations suggest that HA-mediated differences between avian and cattle-associated variants may involve changes in receptor-binding-site geometry rather than a single canonical receptor-switch substitution. To investigate this possibility, we predicted the HA trimer structures of all unique variants using AlphaFold3.01 (Supplementary Fig.6) and mapped phenotypic diversification across avian- and cattle-associated lineages (Supplementary Fig.7). Cattle-associated variants (V6–V10) showed greater structural divergence from the avian lineage A variants, including pronounced changes in the receptor-binding site (Supplementary Fig.7, Supplementary Table 1). Differences around the receptor-binding pocket most clearly separated the two lineages: lineage B diverged more strongly than lineage A, with the largest changes occurring in recent cattle variants. Lineage A variants retained a slightly more open pocket tip (Δd130−190), whereas cattle-associated lineage B variants clustered around a shorter tip with greater lateral opening (Δd150−220) (Supplementary Table 1, Supplementary Fig.7). These observations suggest lineage-specific remodeling of the receptor-binding pocket that could influence HA stability or receptor engagement. This remodeling reflects the combined structural effect of naturally occurring amino-acid substitutions, rather than the absence of mutations; the distinction is that these effects are distributed across the HA head and binding pocket instead of being explained by a single canonical receptor-switch mutation.

To assess the energetic consequences of these structural differences, we compared predicted folding stability across lineages. Mutations in cattle-associated lineage B were predicted to impose larger stability penalties than those in lineage A, with the strongest effects among variants detected in cattle (Supplementary Fig.8, Supplementary Table 1). Estimates of pH-dependent stability showed a similar pattern: lineage A variants tended toward slightly greater stability under acidic conditions, whereas lineage B variants shifted toward neutral or destabilizing values, again with the largest changes in the cattle-associated subset (Supplementary Fig.8). In contrast, predicted receptor-binding energies, estimated from benchmarked Rosetta flex ddG calculations on α2,3- and α2,6-sialoside complexes, remained tightly centered near the reference virus for both α2,3- and α2,6-linked sialosides, with only a few modest outliers. Because most predicted differences fell within a narrow range (~1 REU), these estimates were not interpreted as evidence for lineage-level shifts in receptor preference.

**Figure 3.**
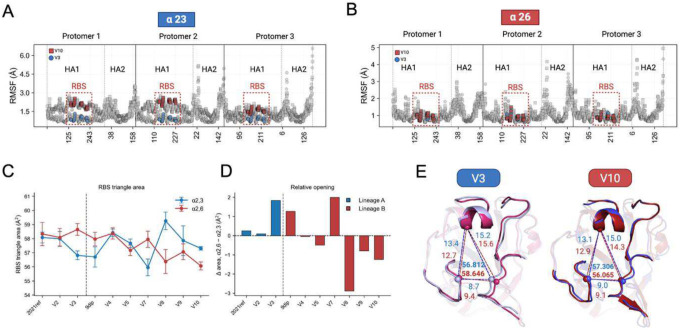
Remodeling of receptor-binding site geometry during the avian-to-cattle transition. (A, B) Backbone root mean square fluctuation profiles for each residue in HA trimer from both the avian (V3) and cattle (V10) lineage variant bound to α2,3 (A) or α2,6 (B) sialosides. The receptor binding regions are highlighted by red-dashed boxes and residues are shown for Lineage A V3 (blue circles) and Lineage B V10 (red squares). (C) Mean receptor binding site triangle area across variants for α2,3 (blue) and α2,6 (red), averaged over 3 protomers in 3 replicate simulation runs. (D) Relative opening quantified as Δarea=area(α2,6)−area(α2,3) for each variant. (E) Representative receptor binding site opening for V3 and V10 comparing α2,3 (blue) and α2,6 (red) geometries. Distances shown are measured in Å.

Molecular dynamics simulations suggested lineage-specific differences in receptor-binding site (RBS) flexibility and geometry that distinguish avian and cattle-associated variants (Fig. 3). Across all simulations, HA trimers remained structurally stable with no evidence of backbone unfolding, indicating that observed differences reflect localized changes in RBS dynamics rather than global structural instability (Supplementary Fig.9). Within the RBS loops, the cattle-associated lineage B variant V10 exhibited greater fluctuations than the avian lineage A variant V3 under α2,3-bound conditions, particularly across the 130-loop, 190-helix, and 220-loop regions that define the receptor-binding pocket (Fig. 3A). In contrast, when bound to α2,6-linked sialosides, both lineages displayed broadly similar fluctuation profiles, suggesting that the enhanced flexibility in lineage B is specific to the avian-type receptor configuration rather than a general increase in protein mobility (Fig. 3B). Consistent with this interpretation, comparisons with the ancestral virus showed that V1 and V3 complexes exhibited slightly greater loop flexibility in α2,6 relative to α2,3 states (Supplementary Fig.10).

We next quantified how these dynamic differences affect receptor-binding site geometry by measuring the area of a triangle defined by Cα atoms at residues 133, 187, and 223, which capture the opening of the binding pocket. The 2021 reference virus (V1) showed minimal dependence on receptor linkage, indicating a relatively stable pocket geometry (Fig. 3C). In contrast, the dominant avian lineage A variant (V3) exhibited a larger receptor-binding site opening when bound to α2,6-linked sialosides, indicating a modest α2,6-favored geometry (Fig. 3C, D). Lineage B variants followed a different trajectory. Early representatives (9DIP and V7) showed slightly larger α2,6 than α2,3 pocket areas, but later cattle-associated variants (V8–V10) reversed this relationship, exhibiting smaller α2,6 openings relative to α2,3 (Fig. 3D, Supplementary Fig.11). Representative structures illustrate these lineage-specific conformations: V3 adopts a wider pocket when accommodating α2,6 glycans, whereas V10 displays a rearrangement of the 130- and 220-loop boundaries that reshapes the binding pocket (Fig. 3E). Together, these analyses support a model in which the receptor-binding site undergoes lineage-specific geometric remodeling during the transition from avian to cattle-associated viruses, while emphasizing that these conformational differences require experimental structural validation.

### Distributed changes in HA receptor engagement occur without canonical receptor-switch mutations

Structural remodeling of the receptor-binding site raises a key functional question: Does this remodeling alter receptor engagement? To address this, we quantified receptor interactions across molecular dynamics generated ensembles using implicit-solvent binding free-energy estimates.

**Figure 4.**
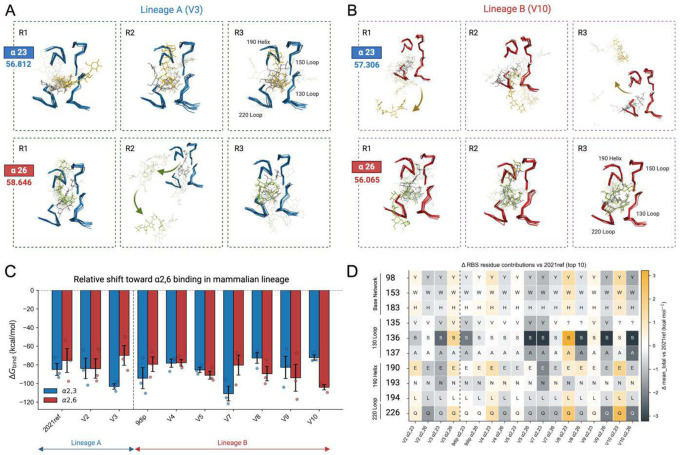
Molecular dynamics and binding energy estimate (MM/GBSA) reveals receptor binding re-tuning relative to the 2021 reference (A, B) Representative snapshots every 100 ns from HA/Sialoside co-complex MD simulation trajectory (top: HA bound to α2,3 and bottom: HA bound to α2,6 sialoside) shown for Lineage A V3 (A) and Lineage B V10 (B) across three replicate simulations (R1–R3). Sialosides are shown as sticks with early-to-late frames colored ash-to-gold for α2,3 amd ash-to-green for α2,6. The first frame and the last frames are shown with stick model. C)Total binding energy for α2,3 and α2,6 sialosides. (D) Heat map of per residue contribution shifts in the top ten RBS residues (relative to 2021 reference) for α2,3 and α2,6.

Trajectory overlays revealed clear lineage-specific conformational differences in how the receptor-binding site accommodates sialoside ligands (Fig. 4A,B). In both avian V3 and cattle V10, receptor interactions remained centered around the binding pocket, although the bound glycans adopted broader conformational distributions. The sialoside occupancy showed occasional partial dissociation events, with more frequent α2,6 losses in V3 and more frequent α2,3 losses in V10 (Fig. 4A, B).

Binding free-energy estimates suggested that these structural differences correspond to relative changes in receptor engagement. Early variants retained a strong energetic preference for α2,3-linked sialosides, consistent with the avian-like binding profile of the 2021 reference virus. This baseline was reinforced in the dominant avian lineage A variant (V3), which strengthened α2,3 binding relative to the ancestral virus (Fig. 4C, Supplementary Figs.12–13). In contrast, variants associated with cattle transmission exhibited a progressive energetic shift toward α2,6-linked sialoside engagement. Although the earliest cattle-associated variant (V7) remained strongly α2,3-favored, subsequent derivatives (V8–V10) showed progressively greater permissiveness for α2,6 engagement in this comparative modeling framework (Fig. 4C).

Per-residue energy decomposition revealed how this transition arises. Rather than being driven by a single adaptive substitution, the energetic landscape of the receptor-binding site was redistributed across multiple residues (Fig. 4D). In early viruses, α2,3 binding was dominated by conserved pocket residues such as 190 and 226. In the cattle-associated lineage, these central contributions weakened, while surrounding residues, including 135, 136, and 226, contributed more strongly to α2,6 binding. Interaction maps from representative molecular dynamics snapshots further showed that V3 contacts remained centered on the sialic-acid moiety, whereas in V10 the interaction network extended along the glycan for both α2,3 and α2,6 linkages (N224, G225, R227) (Supplementary Figs.14–15). In V10, the core sialic-acid network is retained, but contacts spread further along the glycan for both α2,3 (for example K156, D159, K196, Q222, G225) and α2,6 (G143, N187, Q222, R227). Consistent with this redistribution, the largest linkage-dependent shift occurs at the terminal sialic acid: in later dairy-lineage variants (V8–V10), interactions weaken for α2,3 but strengthen for α2,6 relative to the 2021 reference (Supplementary Figs.12, 13). Together, these results suggest that HA receptor engagement can be altered through distributed changes around the binding pocket, even when classical receptor-switch residues like Q226 and G228 remain largely avian-like.

Several methodological limitations should be considered when interpreting these energetic estimates. The modeled ligands represent short linear NeuAc-containing α2,3- and α2,6-linked sialosides and therefore do not capture the full complexity of airway or mammary glycomes including NeuGc-containing bovine mammary glycans. HA glycosylation was not explicitly modeled in these systems. Binding free-energy estimates should therefore be interpreted primarily as comparative metrics across variants within the same protocol rather than absolute affinities, and differences within ~1–2 kcal mol^−1^ should be interpreted cautiously pending experimental validation using full-length glycosylated HA and native glycan repertoires. Finally, this analysis focuses on HA and does not evaluate contributions from other viral genes such as NA or PB2.

## Discussion

Cross-host emergence is commonly framed as a process of progressive optimization, in which viral lineages accumulate adaptive mutations that incrementally increase fitness in a new host [22, 26–30]. Our results instead suggest that HA-mediated cross-host potential may arise not from the most optimized viral lineages, but from those with the greatest HA functional flexibility. Across a large phylogenetic and phenotypic dataset, we show that geographic expansion [1, 5] generates extensive HA phenotypic diversity that is subsequently pruned by selective sweeps, that cattle-associated HA backgrounds can be detected in avian circulation before their enrichment in cattle, and that cattle-associated receptor-engagement patterns can arise without receptor-site substitutions typically associated with mammalian adaptation. Together, these findings suggest that cross-host potential may depend less on peak fitness in any single host than on standing functional breadth that enables viruses to persist and transmit across heterogeneous environments. Importantly, this interpretation does not imply that cattle-associated phenotypes arose without new mutations after spillover; rather, it indicates that standing avian HA diversity and subsequent cattle-associated diversification jointly shaped the predicted HA phenotype landscape. This interpretation is consistent with recent cross-virus phylogenetic evidence that extensive pre-zoonotic adaptation is often not detectably intensified immediately before zoonotic outbreaks, suggesting that emergence can proceed from pre-existing functional capacity rather than prolonged stepwise optimization [19].

Current strategies for evaluating influenza cross-host risk are poorly suited to detect this form of evolutionary potential. Surveillance frameworks largely prioritize the identification of known adaptive markers, implicitly assuming that emergence requires identifiable genetic optimization [27, 31, 32]. Experimental approaches such as deep mutational scanning [33] and targeted mutagenesis [11, 12, 34] provide detailed functional maps, but they interrogate variants in isolation and rarely capture how naturally circulating populations explore phenotype space over time. Likewise, phylogenetic analyses typically track lineage expansion without integrating quantitative measures of protein function, limiting inference about which variants possess latent host-range capacity [33, 35, 36]. As a result, existing approaches may systematically overlook transient or low-frequency variants whose functional breadth enables host shifts, even if those variants are not the most fit within their original host population [21, 37, 38]. Recent large-scale efforts have started to connect sequence evolution to population-level immune phenotypes for H5, including high-resolution antigenic maps and at-scale computational antibody–HA binding surveys [39, 40]. However, phenotyping of HPAI is hard to scale because live-virus experiments require high-containment facilities, increasing cost and limiting throughput [41, 42].

Here, we present an integrated framework that directly addresses these gaps by linking naturally occurring genetic variation to quantitative phenotypes across evolutionary, structural, and population scales. Despite the emergence of a cattle-associated lineage, receptor preference remains largely avian-type. Because Lineage A and Lineage B are defined from the HA phylogeny and predicted HA phenotype trajectories, they should be interpreted as HA-centered descriptors rather than whole-genome genotype labels such as B3.13 or D1.1. Instead, the lineage split reflects subtle, coordinated tuning of the HA head: the cattle-associated lineage B trends toward a slightly more basic, more positively charged HA1, modest reshaping of the receptor-binding pocket (a slightly shorter apex with wider flanks), and lower predicted stability, consistent with recent structural and stability observations [43–45]. However, receptor preference remains largely avian-type, and the main change we observe is a gradual shift in how the pocket supports binding, making α2,6 engagement somewhat more permissive in later dairy-lineage B variants, without a full receptor switch [6, 44, 46, 47].

This HA-centered analysis should not be interpreted as a complete model of host range or pandemic risk. Cross-species transmission also depends on neuraminidase balance, polymerase compatibility, innate immune evasion, tissue tropism, exposure ecology, and onward transmission, which were not measured here. In addition, the structural phenotypes reported here are model-derived and require experimental validation by approaches such as glycan binding assays, glycan arrays, crystallography, or cryo-EM. However, these models showed high structural-confidence metrics, were anchored to experimentally solved HA–sialoside crystal structures, and were further evaluated by RMSD-based structural comparisons, Rosetta relaxation/benchmarking, and replicate MD stability analyses. Thus, these results identify predicted HA features that may warrant targeted experimental follow-up, but they do not assign zoonotic risk to individual variants.

By coupling large-scale genomic sampling with structural modeling and predicted thermodynamic inference, our approach identifies functionally credible variants without relying on predefined adaptive markers. Mapping these phenotypes onto time-resolved phylogenies further enables explicit measurement of how phenotypic diversity emerges, persists, and is pruned during lineage expansion. In doing so, the framework shifts risk assessment from detecting signatures of prior adaptation to quantifying latent functional flexibility, providing a scalable strategy to identify variants with cross-host potential before they rise to dominance.

These observations are compatible with ecological fitting, in which host transitions may draw on pre-existing functional breadth within viral populations rather than arising solely through stepwise adaptive mutation. Further investigation into the role of non-adaptive phenotypic plasticity in ecological fitting is critical to better understand the underlying mechanisms of viral host transitions and cross-host emergence. Combining large-scale molecular epidemiological analyses with experimental investigations and ecological modeling will be critical for evaluating emergence risk and informing preparedness efforts.

Although our analyses do not directly demonstrate ecological fitting experimentally, several independent lines of evidence support the biological plausibility of this interpretation [13, 44, 47, 48]. Recent experimental studies of H5N1 viruses circulating in dairy cattle show that hemagglutinin retains predominantly avian-type receptor specificity while nevertheless supporting mammalian infection and transmission, indicating that classical receptor-switch mutations are not required for host transition [47]. These studies also emphasize that cattle-associated adaptation is mutation-driven and can involve mechanisms outside the NeuAc α2,3/α2,6 receptor models used here, including polymerase adaptation and HA substitutions such as D104G and V147M that enhance engagement of NeuGc-containing bovine receptors [49]. In our dataset, D104G is present among later cattle-associated variants and the 130-loop substitution V147M occurs in V8 and V10, but our receptor models do not directly test NeuGc-mediated binding. Structural and biochemical analyses further demonstrate that alterations outside the traditional receptor-binding site can modulate receptor engagement by altering binding-site flexibility and glycan interaction networks [18, 48, 50, 51]. These observations are consistent with our population-scale findings that cattle-associated phenotypes can arise through distributed structural and energetic changes rather than discrete adaptive substitutions. Our results therefore do not claim to demonstrate ecological fitting directly, but instead provide population-level evidence that viral emergence can draw on standing phenotypic breadth within circulating diversity, a prediction that is increasingly supported by experimental studies of contemporary H5N1 lineages [43, 48].

Our study establishes an H5N1 HA-focused framework for linking naturally circulating sequence variation to predicted protein phenotypes across evolutionary time, revealing that predicted cross-host-relevant HA variation can reflect both sequence change and the distribution of accessible functional states within circulating populations. By showing that cattle-associated HA phenotypes can emerge without classical receptor-switch substitutions, we challenge the assumption that cross-host-relevant features are detectable solely through canonical genetic markers. Rather than viewing emergence as the endpoint of stepwise adaptation, our results suggest that some viral populations may already contain standing reservoirs of HA phenotypic flexibility that are not fully captured by sequence markers alone. This perspective reframes spillover-associated HA phenotypes as features that can be investigated through population-scale exploration of predicted functional space, rather than as changes visible only through single adaptive mutations. Integrating evolutionary dynamics with quantitative phenotype inference, therefore, provides a framework for prioritizing HA backgrounds and receptor-binding-site changes that merit targeted experimental follow-up before epidemiological signals become visible.

## Methods

### Data collection and sequence preprocessing

Complete hemagglutinin (HA) coding sequences and metadata for U.S. H5N1 clade 2.3.4.4b viruses collected from December 2021 to 6 May 2025 were downloaded from the GISAID EpiFlu^™^ database [52]. Nucleotide sequences were aligned with MAFFT v7.505 [53] within Seqotron v1.0 [54] and were trimmed to the HA open reading frame (ORF). Sequences that did not cover the full ORF or contained more than 10% ambiguous nucleotides or gaps were removed using an in-house Python script. After these steps, the dataset contained 13,000 HA nucleotide sequences. For records with incomplete collection dates, we imputed the midpoint of the reported month or the midpoint of the reported year. For isolates with incomplete dates collected between January 1 and May 6, 2025, we imputed the midpoint of that window.

### Time-scaled phylogeny and phenotype tip-mapping

We inferred a maximum-likelihood tree for all 13,000 U.S. H5N1 2.3.4.4b HA (DNA) sequences using IQ-TREE 2 [55]. We identified and excluded temporal outliers using root-to-tip regression in TempEst [56]. The tree was converted to a time-resolved phylogeny with TreeTime [57]. To map sequence-level phenotypes onto this tree, we translated the curated nucleotide sequence dataset to amino acids and collapsed identical amino-acid sequences, yielding 2,195 unique HA variants, or “variant classes”, that represent all non-redundant U.S. clade 2.3.4.4b sequences circulating during the period of the study. Briefly, we built a lookup table from aligned HA amino acid sequence to its representative accession and then scanned the full 13,000-tip HA amino acid alignment. Each tip was assigned to a variant class if its aligned HA sequence matched one of the 2,195 unique sequences exactly. This created a one-to-many relationship of representative variant to all isolates carrying that HA sequence. Variant frequencies were obtained by counting the number of isolates across all tree tips that mapped to each variant class. The “top 10” variants were defined as the ten variant classes with the highest tip counts (most frequently observed amino acid sequences in the U.S. dataset). For tree visualizations, we used the baltic library (available at https://github.com/evogytis/baltic) to read the time-scaled tree (timetree.nexus). We also mapped all the phenotypes using the baltic library.

Some variants had incomplete sequence information because the analysis was based on a trimmed HA structural core, defined by the 9DIP coverage, with N- and C-terminal residues and the cleavage region excluded. In addition, some sequences contained ambiguous amino acids, denoted as “X.” To address this, we performed maximum-likelihood ancestral reconstruction using IQ-TREE 2 [55] on the protein alignment, which generated both a phylogeny and a node-state file (.state). We then reconstructed ancestral node sequences, identified the parental node for each tip, and replaced each ambiguous “X” with the corresponding parental amino acid when the posterior probability was at least 0.70. If no confident ancestral state was available, the global consensus amino acid at that position was used instead. Thus, whenever sequence information was missing or ambiguous, residues were imputed from the local most recent common ancestor or consensus sequence. Before trimming and cleaning, 371 of 2,195 unique HA amino-acid variants contained at least one ambiguous residue, totaling 676 ambiguous residues across 297 alignment positions. After trimming to the 9DIP-covered structural core and applying the cleaning/imputation workflow, the final 2,127-variant structural-core alignment contained no ambiguous residues.

### AlphaFold-3 structural modeling

To capture structural variability across clade 2.3.4.4b, we predicted 3D structures for every unique HA amino acid sequence using AlphaFold-3.01 [58]. To assess the quality of the predicted structures, we calculated Predicted Local Distance Difference Test (pLDDT) scores; higher values correspond with higher confidence in the local structure [59]. To compare structures to each other, we measured backbone similarity using root-mean-square deviation (RMSD), which reports the average distance between matched backbone atoms after the proteins are overlaid using MDAnalysis [60]. Briefly, we compared overall HA protein shape by overlaying each pair of trimers using their shared backbone Cα atoms (a standard Kabsch superposition), matching the same chain and residue positions, and then recording the backbone RMSD. We then repeated the comparison focusing only on the receptor-binding pocket region (the 130-loop, 150-loop, 190-helix, and 220-loop) to measure how much that pocket differs between variants. Finally, to describe how “open” the pocket is in a simple geometric fashion, we calculated the center point (centroid) of each segment and measured distances between them across the pocket tip and sides (d130–190, d130–220, d150–220).

#### Structural model preparation for Rosetta

Structural modeling was based on two high-resolution HA trimer crystal structures from A/Texas/37/2024 deposited in RCSB Protein Data Bank [61] as 9DIP (α2,3-bound; 2.32 Å) [13] and 9DIO (Q226L mutant, α2,6-bound; 2.70 Å) [13]. 9DIP was selected as the primary modeling template due to its superior resolution, with 9DIO serving as a reference. To correct glycan inconsistencies, the complete five-monosaccharide moieties α2,3-sialyllactose chain (LSTa: Neu5Acα2−3Galβ1−3GlcNAcβ1−3Galβ1−4Glc) was extracted from a fully occupied protomer in 9DIP and placed into the deficient receptor binding pockets using PyMOL [62]. For α2,6-bound modeling, the full five-monosaccharide moieties sialyllactose chain (LSTc: Neu5Acα2−6Galβ1−4GlcNAcβ1−3Galβ1−4Glc) from 9DIO (chain G) was extracted and placed onto all three binding pockets of 9DIP using PyMOL after removing the α2,3-sialyllactose from all three chains with careful alignment based on the reference 9DIO. These trimer structures with three five-monosaccharide moieties (both for α2,3 and α2,6) at each receptor binding pocket were then exported for further use. PDB files were then cleaned using Python scripts to remove non-structural records (e.g., ANISOU, CONECT, REMARK) then residues were renumbered sequentially from 1 to 1488 (three protomers × 496 residues). To optimize compatibility with Rosetta’s cartDDG protocol [63], the HA trimer was merged into a single pseudo-monomeric chain while retaining trimeric coordinates. Sialosides were preserved as separate chains at the end of the PDB file. These cleaned models served as the starting templates for all subsequent Cartesian relaxation, mutation modeling, ΔΔG calculations, and molecular dynamics simulations.

#### Cartesian relaxation of HA-Sialic acid complexes

All HA trimer–sialic acid complex structures were refined by energy minimization using Rosetta’s Cartesian-relaxation protocol [63]. We performed this refinement using Rosetta’s beta_nov16_cart scoring function, which is the energy model Rosetta uses to judge whether a structure is physically reasonable. It includes improved treatment of how atoms interact through water/solvation effects, electrostatic interactions, and van der Waals contacts. For a subset of complexes, we also ran the same refinement using ref2015_cart as a comparison benchmark. Because relaxation can end in slightly different local best energy minimized structures, depending on the starting path, we generated 20 independent refined models for each complex (−nstruct 20) and then selected the lowest-energy refined structure for the next analyses, including stability (ΔΔG) calculations and molecular dynamics simulations (Supplementary Fig.16). The relaxation itself followed a staged protocol (cart2.script) where it starts with stronger restraints and more conservative settings to avoid unrealistic jumps, then gradually reduces steric repulsion scaling and loosens coordinate constraints so the structure can settle into a more natural, low-energy conformation. Carbohydrate stereochemistry, linkages, and naming were preserved using Rosetta flags [64] (-ideal_sugars, -auto_detect_glycan_connections, -maintain_links, -include_sugars, -alternate_3_letter_codes pdb_sugar). Although -write_pdb_link_records and -write_glycan_pdb_codes restored residue names (e.g., SIA, GAL, NAG), glycosidic bonds remained inconsistently visualized in PyMOL, necessitating manual correction of glycan renaming using a custom Python script.

### Mutation library construction

To model all naturally occurring mutations, we aligned the reference 9DIP HA sequence with 2,195 unique U.S. clade 2.3.4.4b HA sequences. Amino acid sequences were aligned with MAFFT, trimmed to remove unresolved regions (signal peptide, polybasic cleavage site, C-terminal tail), and converted to the pseudomonomer 1–1488 numbering used in the Rosetta models. Where sequence information was ambiguous or missing, we reconstructed the parental node or global consensus as described in the “AlphaFold-3 structural modeling” section to impute residues. Finally, for each HA variant, a Python script compared the sequence to the 9DIP reference, enumerated standard amino acid mismatches, and generated Rosetta .mut files (for cartddg2020) and matched .resfile specifications (for flex ddG). Mutations were applied symmetrically to the trimer by offsetting positions by +496 and +992 in the chain.

#### Rosetta Cartesian ΔΔG (cartddg2020) mutation scanning

Following structure relaxation and mutation file generation, we applied Rosetta’s cartddg2020 protocol [63] to estimate ΔΔG changes upon mutations across three structural states: (i) apo HA-only (which also gave the estimation of HA thermodynamicstability), (ii) HA−α2,3-sialyllactose (3′SLN) complex, and (iii) HA-α2,6-sialyllactose (6′SLN) complex. For each of the unique HA variants, corresponding .mut files were used to introduce mutations symmetrically. Five minimization and repacking iterations that allowed local backbone flexibility were performed per mutation (-ddg::iterations 5) within a three-residue window (-ddg::bbnbrs 3) while maintaining minimization constraints.

Wild-type and mutant energy scores (Rosetta Energy Units, REUs) were averaged over replicates for each condition. To treat sialic acid properly, we used the same flags selected for cartesian relaxation. To quantify binding effects, we used a flex ddG format [65] thermodynamic cycle, treating HA as the mutating partner and keeping sialyllactose fixed. This allowed us to calculate ΔΔG_bind values which reflect the mutation’s impact on receptor binding affinity. The flex ddG protocol equation is as follows:

$$\Delta \Delta {\text{G}}_{\text{bind}}=\Delta {\text{G}}_{\text{bind}}^{MUT}-\Delta {\text{G}}_{\text{bind}}^{WT}\;=\left(\Delta {\text{G}}_{\text{complex}}^{MUT}-\Delta {\text{G}}_{\text{partner}\;A}^{MUT}-\Delta {\text{G}}_{\text{partner}\;B}^{MUT}\right)\;-\left(\Delta {\text{G}}_{\text{complex}}^{WT}-\Delta {\text{G}}_{\text{partner}\;A}^{WT}-\Delta {\text{G}}_{\text{partner}\;B}^{WT}\right)$$

For our case, the partner A is HA and partner B is sialoside, and, therefore, binding free energy (ΔG_bind) for each state was computed as:

$$\Delta {\text{G}}_{\text{bind}}={\text{G}}_{HA+\text{Sia}}-{\text{G}}_{HA}-{\text{G}}_{\text{Sia}}$$

The mutation-induced change in binding energy, or ΔΔG_bind, was computed as the difference between mutant and wildtype binding energies:

$$\Delta \Delta {\text{G}}_{\text{bind}}=\Delta {\text{G}}_{\text{bind}}^{MUT}-\Delta {\text{G}}_{\text{bind}}^{WT}$$

Substituting the expression for ΔG_bind, this becomes:

$$\Delta \Delta {\text{G}}_{\text{bind}}=\left({\text{G}}_{HA+\text{Sia}}^{MUT}-{\text{G}}_{HA}^{MUT}-{\text{G}}_{\text{Sia}}\right)-\left({\text{G}}_{HA+\text{Sia}}^{WT}-{\text{G}}_{HA}^{WT}-{\text{G}}_{\text{Sia}}\right)$$

Because ${\text{G}}_{\text{Sia}}$ is the same across all systems and cancels out, the equation simplifies to:

$$\Delta \Delta {\text{G}}_{\text{bind}}=\left({\text{G}}_{HA+\text{Sia}}^{MUT}-{\text{G}}_{HA}^{MUT}\right)-\left({\text{G}}_{HA+\text{Sia}}^{WT}-{\text{G}}_{HA}^{WT}\right)$$

#### pH-dependent stability estimation

To estimate how HA folding stability changes with pH, we used FoldX (v5.1) [66] in two stages. First, each starting HA structure was optimized with RepairPDB at pH 7.0, ionic strength 0.05, and 298 K to remove local steric clashes and standardize side-chain conformations prior to mutation modeling. Second, for each HA variant, we ran BuildModel across a predefined pH panel (4.5, 5.0, 5.3, 5.5, 5.7, 6.0, 6.5, 7.0) at 298 K and ionic strength 0.05, using multiple independent FoldX runs per variant (NRUNS = 3). For each variant and pH, we extracted the total energy and summarized the mean across runs as a pH-specific stability proxy (ΔΔG-like total-energy change). To summarize pH sensitivity as a single trait, we computed acid_mean_minus_neutral, defined as the mean stability change at selected acidic points minus the neutral condition:

$$\text{acid\_mean\_minus\_neutral}=\text{mean}\left(\Delta {\text{G}}_{pH=5.7,5.5,5.3}\right)-\Delta {\text{G}}_{pH=7.0}$$

Positive values indicate variants that become relatively more destabilized under acidic conditions compared with neutral pH, while negative values indicate comparatively improved stability at acidic pH.

#### Rosetta flex ddG binding energy calculations

We computed backrub-based interface ΔΔG values using a RosettaScripts implementation of flex ddG [65] with Rosetta and the full-atom beta_nov16 energy function. Single or combinatorial mutations were specified via .resfiles derived from the mutation library described in the earlier section. We restricted design to the target mutation sites and defined a local “neighbor shell” as all residues within 8 Å of any mutated position and their immediate sequence neighbors. Residues in this shell were allowed to repack; all other residues were kept fixed. For each mutation, we applied Cα coordinate restraints and first minimized the wild-type complex (backbone and side chains). We then ran a Monte Carlo backrub protocol (35,000 trials) on the neighbor shell to generate an ensemble of local backbone conformations. For each saved conformation, we repacked and minimized the wild-type complex, recorded its interface energy, introduced the specified mutation, repacked and minimized the same neighbor shell, and recomputed the interface energy with *InterfaceDdGMover*, while treating HA as the mutating partner. Interface ΔΔG values were calculated as mutant minus wild-type interface energy and averaged over the backrub ensemble. All calculations preserved carbohydrate residues *(-include_sugars, -alternate_3_letter_codes pdb_sugar, -maintain_links)*. Jobs were run on an HPC cluster as SLURM arrays, with one MPI rosetta_scripts task per mutation .resfile. We additionally tested a variant in which Cartesian relaxation was applied before flex ddG (“flexddg_wCrelax”) versus direct application to the complexes (“flexddg_woCrelax”).

#### Benchmarking Rosetta protocols against experimental receptor-binding data

To calibrate Rosetta ΔΔG predictions to experimentally measured receptor binding, we benchmarked multiple Rosetta protocols against the A/Texas/37/2024 HA mutants characterized by Lin et al. [13]. The benchmark set comprised the wild type and five engineered variants- Q226L, G228S, N224K/Q226L, N224K/Q226L/G228S, and E190D, which span the avian-to-human receptor switch (α2,3 vs α2,6) and loss-of-binding phenotypes described in their study [13]. For structural inputs, we used: (i) the 9DIO Q226L−α2,6 complex (9DIO complex), (ii) a reverse-mutated 9DIO L226Q model, (iii) 9DIP α2,3-bound, (iv) 9DIP with grafted α2,6 sialoside (from 9DIO), and (v) the corresponding apo HA-only states. For each configuration, we ran a panel of Rosetta protocols including cartddg2020 with backbone-flexibility radii “*bbnbrs* 1–3” under “*beta_nov16_cart*” or “*ref2015_cart*”, with either globally/locally constrained or unconstrained relaxation, and flex ddG with and without prior Cartesian relaxation (Fig S16). Experimental equilibrium dissociation constants (K_D) for α2,3-linked (3SLN-L / LSTa) and α2,6-linked (6SLN-L / LSTc) ligands were taken from Lin et al. [13] and converted to experimental ΔΔG values using $\Delta \Delta {\text{G}}_{\text{exp}}=RT\;\text{ln}\left(\frac{K_{D}^{\text{mut}}}{K_{D}^{\text{mut}}}\right)$, where R = 0.001987 kcal mol^−1^ K^−1^ and T = 298 K. We then used a custom Python script to merge predicted and experimental ΔΔG values, and computed Pearson and Spearman correlations, 95% confidence intervals (Fisher z transform), and root-mean-square error (RMSE) for each protocol. These analyses identified the protocol variants that best recapitulated the experimental gain- and loss-of-binding phenotypes for the calibration mutants and guided the choice of cartddg2020/flex ddG settings used for the large-scale 2.3.4.4b mutation scan. The flex ddG protocol with prior Cartesian relaxation best matched measured α2,3/α2,6 binding (r = 0.73; RMSE = 3.3 kcal mol^−1^), outperforming cartddG and unrelaxed flex ddG (Supplementary Fig.8A).

#### Structure preparation for molecular dynamics simulations

While Rosetta protocol provides high-throughput thermodynamic estimates for HA mutations, its static treatment of sialic acid and limited backbone flexibility restricts dynamic interpretation. To overcome these limitations, we incorporated explicit-solvent molecular dynamics (MD) simulations. We ran all-atom MD simulations with AmberTools24 [67] and Amber24 [68] on HA-sialoside complexes on top ten representative variants. We first standardized all Rosetta-relaxed PDB structures (HA+3′SLN and HA+6′SLN) using pdb4amber [68], which remedied the formatting and atom name issues. Next, all plausible disulfide bonds between cysteine residues in the HA were assigned. To finalize the PDBs for Amber compatibility, another custom python script converted all HETATM records to ATOM, inserted TER lines between individual glycan residues (e.g., ROH, 4YA, 3LB, 0SA), and removed redundant CONECT entries. Manual editing was necessary to fix glycan topologies and sialic acid chemistry, guided by GLYCAM06j-1 force field conventions [69]. Key updates included renaming residues and atoms to match GLYCAM naming convention (e.g., SIA → 0SA, GAL → 3LB, NAG → 4YA). The reducing ends on the sialoside were capped with hydroxide (ROH). Finally, atom and residue indices were renumbered, and all bond records were updated, visualized in PyMOL [62] and documented as glycosidic bond file before MD system setup.

#### Molecular dynamics and post-MD trajectory analysis

Complexes that were prepared in the previous step were parameterized with the ff19SB force field [70] for HA and GLYCAM_06j-1 [69] for sialosides 3′SLN and 6′SLN, solvated in an octahedral TIP5P water box [71] (10 Å buffer) and neutralized with counterions using tleap, available in the Amber suite [67]. We followed a reproducible 10-step protocol recommended by Roe and Brooks [72] and implementation provided in GLYCAM-Web (https://github.com/GLYCAM-Web/MD_Utils/tree/actual/protocols/RoeProtocol). It includes sequential steps of minimization, restrained heating, and pressure equilibration, with restraints gradually reduced from 500 kcal/mol/Å^2^ to none. Energy minimization followed a four-stage protocol of 10,000 cycles each (steepest descent followed by conjugate gradient), starting with positional restraints of 5.0 kcal·mol^−1^·Å^−2^ on all non-water heavy atoms and gradually reducing the restraint weight to 2.0 and 0.1 kcal·mol^−1^·Å^−2^ before a final unrestrained minimization. Then, the systems were heated to 300 K under NVT conditions with 5.0 kcal·mol^−1^·Å^−2^ positional restraints on solute heavy atoms. This was followed by a series of short NPT segments (1 bar) at 300 K with a Langevin thermostat $\left(\gamma =5{\text{ps}}^{-1}\right)$, gradually reducing restraints on solute heavy atoms from 1.0 to 0.5 kcal·mol^−1^·Å^−2^ and then restricting the remaining restraints to protein backbone and glycan linkage atoms, before a final unrestrained NPT equilibration. All simulations used a nonbonded cutoff of 8 Å, SHAKE [73] constraints on bonds to hydrogen (bond constraint control (ntc)= 2, force evaluation control (ntf) = 2), and particle mesh Ewald [74] for long-range electrostatics. Production MD was run in the NPT ensemble at 300 K and 1 bar using a Langevin thermostat and isotropic pressure coupling, with a 2 femtosecond (2fs) time step and the same nonbonded and SHAKE settings. Coordinates, energies, and pressure were written every 100 picoseconds (ps). For each HA system, we simulated both α2,3- and α2,6-bound complexes in triplicate for 500 ns production runs each. The set was comprised of the A/AmericanWigeon/SouthCarolina/22-000345-001/2021 HA (designed using the 9DIP scaffold), the 9DIP crystal complex (with α2,3- and α2,6-bound) and eight other most circulating variants, each simulated with both linkages (α2,3- and α2,6). In total, this yielded 30 μs of explicit-solvent trajectory data (60 independent 500-ns simulations) used for downstream conformational and interaction analyses.

Post-MD analyses were conducted using cpptraj [75] and custom Python pipelines to quantify structural dynamics and receptor interactions. Stability metrics, including RMSD and root mean square fluctuation (RMSF) were extracted to monitor convergence and flexibility. Principal component analysis (PCA) on Cα atoms was used to characterize dominant motions, and the densest conformational state was extracted via kernel density estimation (KDE) for representative interaction profiling with PLIP [76]. To characterize time-resolved HA-sialoside geometry and contacts, we additionally extracted every snapshot (1000 frames per run) from each trajectory. Finally, for each production trajectory, we used cpptraj to compute RBS geometry metrics separately for all three protomers of the HA trimer. Specifically, after auto-imaging and backbone RMS fitting, we defined 130 and 220-loop segments in each protomer and calculated (i) a mouth width as the center-of-mass distance between the 130 and 220-loop segments and (ii) three Cα−Cα distances between anchor residues 133, 187 and 223, which span the RBS apex and flanks. The three anchor distances were converted to a per-frame RBS triangle area using Heron’s formula, and area trajectories were first summarized per protomer and then averaged across the three protomers to obtain a single RBS opening metric per MD run. For structural visualization and contact analysis, we then identified, for each run, the protomer-1 frame whose triangle area was closest to that run’s mean area and extracted a backbone- and glycan-containing PDB (protomer 1 + its attached RBS glycan) from the aligned trajectory using cpptraj.

#### Binding free energy estimation using MM/GBSA

To quantify the effect of naturally occurring HA mutations on receptor-binding energies in a dynamic context, we employed MM/GBSA (Molecular Mechanics/Generalized Born Surface Area) [77] with per-residue decomposition. Water molecules and counterions were stripped (strip_mask=‘:WAT,Na+,Cl-’). Solvation was treated with the igb=2 Generalized Born model (GB-OBC2) at 0.15 M ionic strength to approximate physiological salt conditions. For each snapshot, MM/GBSA energies were evaluated for the complex, HA (receptor), and sialoside (ligand), and the binding free energy was estimated as

$$\Delta {\text{G}}_{\text{bind}}={\text{G}}_{\text{complex}}-{\text{G}}_{\text{receptor}}-{\text{G}}_{\text{ligand}}$$

Each free energy term was decomposed into molecular mechanics energy, solvation energy, and an optional entropic contribution (not included in this study due to computational cost and convergence issues across large variant sets),

$$G={\text{E}}_{MM}-{\text{G}}_{\text{solvation}}=\left({\text{E}}_{\text{int}}+{\text{E}}_{\text{ele}}+{\text{E}}_{\text{vdW}}\right)+\left({\text{G}}_{\text{polar}}+{\text{G}}_{\text{non–polar}}\right)$$

Here, $E_{MM}$ is the gas-phase molecular mechanics energy (bonded + van der Waals + electrostatics) and $G_{\text{solvation}}$ is the polar + nonpolar solvation free energy from the GB model and a surface-area term, respectively. We also obtained per-residue contributions using pairwise decomposition (idecomp=2).

#### Sequence-based phenotype prediction

For each of the 2,195 unique HA sequences, we computed a panel of sequence-derived phenotype metrics using Biopython’s ProtParam module [78] and custom Python script. A representative early U.S. clade 2.3.4.4b HA sequence, A/AmericanWigeon/SouthCarolina/22–000345-001/2021, was used as the reference for mutation-based metrics. All metrics described below were calculated on the gap-stripped amino acid sequence.

Global biophysical properties were calculated for the full HA sequence. The grand average of hydropathy (GRAVY) was computed as

$$\text{GRAVY}=\frac{1}{L}\sum_{i=1}^{L}h_{i}$$

where L is the sequence length and ${\text{h}}_{\text{i}}$ is the Kyte–Doolittle hydropathy index of residue i. The isoelectric point (pI) was estimated by ProtParam using standard residue pKa values and iterative adjustment of pH.

The aliphatic index was calculated as

AI=100*[X(Ala)+2.9X(Val)+3.9(X(Ile)+X(Leu))]

where X denotes the mole fraction of each residue type. These metrics were also computed separately for the HA1 (globular head) and HA2 (stem) domains.

Charge-related metrics included the fraction of positively and negatively charged residues and the net charge at pH 7. The fraction of positively charged residues was defined as

$$\text{pos\_frac}=\frac{n(K)+n(R)}{L}$$

and the fraction of negatively charged residues as

$$\text{neg\_frac}=\frac{n(D)+n(E)}{L}$$

where n denotes the count of each amino acid in the sequence, K, R, D, and E denote lysine, arginine, aspartic acid, and glutamic acid, respectively, and L is sequence length. Net charge at pH 7 was estimated by ProtParam using Henderson-Hasselbalch-type calculations for all ionizable side chains and termini. Net charge was also calculated for the full-length HA and separately for HA1 and HA2.

Secondary-structure propensities were estimated using ProtParam’s composition-based predictor, which partitions residues into helix-, sheet-, and turn-favoring categories. This yielded three values: helix_frac, sheet_frac, and turn_frac, corresponding to the fraction of residues predicted to favor α-helix, β-strand, or turn conformations, respectively.

#### Phenotypic diversity estimation

To quantify how broadly each continuous phenotype was distributed through time, we discretized phenotype values into quantile-based bins and summarized the resulting bin occupancy using an effective-bin diversity index (Shannon Hill number, q=1). For each phenotype metric, we first computed global bin edges using one value per unique HA variant to avoid oversampling bias from repeatedly sequenced isolates. Bin edges were defined using quantile binning, targeting 5 bins; when a metric contained too few unique values, duplicate quantile edges were automatically collapsed yielding fewer than 5 bins for that metric. Each observation was then assigned a bin index using a right-closed edge rule, with missing/non-finite values excluded from bin counts.

Within each time period (quarterly aggregation), we computed bin counts and converted them to proportions. Effective bins were calculated as the Shannon Hill number:

$$H_{2}=\sum_{i}p_{i}\;{log}_{2}\left(p_{i}\right),\;\text{EffectiveBins}=2^{H_{2}}$$

which corresponds to the number of equally frequent bins that would produce the observed entropy.

To control for unequal sampling intensity across time, we used rarefaction-based bootstrapping (without replacement) for periods meeting a minimum sample size threshold (MIN_PERIOD_N = 25). For each analysis scope and basis, we set a common rarefaction depth:

$$n_{\text{rarefy}}=\text{min}\left\{n_{t}|n_{t}\geq 25\right\}$$

where ${\text{n}}_{\text{t}}|{\text{n}}_{\text{t}}$ is the number of items in a time period. For each eligible period and metric, we drew 600 random subsamples of size ${\text{n}}_{\text{rarefy}}$ without replacement (BOOTSTRAP_ITERS = 600, SEED = 7), recomputed EffectiveBins for each draw, and summarized results as the mean and 95% interval (2.5th–97.5th percentiles). Periods with <25 were not rarefied and were reported/visualized using raw (non-rarefied) values only.

All diversity calculations were performed on sequence-basis, where each isolate contributed one observation. Phenotype values that could not be computed were treated as missing and were not imputed. During phenotypic diversity analysis, missing values were excluded from bin assignment. Rarefaction-based bootstrapping was performed only when a time period contained at least the required number of non-missing observations, and subsampling was performed without replacement.

For the top 10 variants, we quantified phenotypic shifts for each variant as the difference from the V1 reference, and summarized divergence using a centroid-proximity approach. For each phenotype (and for each domain-specific metric, for example HA, HA1, and HA2, treated separately), we first learned a “no-change” band from the full dataset after outlier mitigation by clipping extreme values to the 1st–99th percentile range. Variants falling within this no-change band were classified as unchanged and shown in light blue. For variants outside the no-change band, we defined two reference anchors from the sequence-weighted dataset: the median phenotype value for Lineage A (avian-like anchor) and the median phenotype value for Lineage B restricted to domestic mammal (DM) sequences (cattle-like anchor). Each variant was then assigned as avian-like or cattle-like by comparing its absolute distance to these two medians (closer to the Lineage A median, avian-like; closer to the Lineage B DM median, cattle-like) and visualized with color (blue for avian-like, red for cattle-like). Color intensity represents effect size using magnitude bins derived from the full distribution of absolute shifts for that phenotype.

Across the manuscript we use three complementary color encodings to visualize phenotype change in different ways. First (Fig. 1), tree tips are colored by an overall lineage-likeness score that summarizes the multi-trait phenotype atlas: per-phenotype deviations are binned, assigned a sign by proximity to avian-like versus cattle-like anchors, and then averaged across phenotypes to yield a single centered scale (blue, avian-like; red, cattle-like; white, reference-like/no-change). Second (Fig. 2), colors represent lineage-median proximity: values are mapped to how close each variant is to the Lineage A median versus the cattle (Lineage B, domestic-mammal) median, with the 2021 reference shown in light blue for orientation. Third (Supplementary Fig. 1), we provide reference-centric views of effect size: inSupplementary Fig. 1, variants are discretized into a five-level scheme (gray for minimal change from the reference, increasing blue for negative shifts, increasing red for positive shifts), whereas in subsequentSupplementary trees we use a continuous diverging scale where sign indicates direction (blue negative, red positive) and color intensity reflects the magnitude of deviation from the reference.

## Supplementary Files

This is a list of supplementary files associated with this preprint. Click to download.

- reference2021DNAseq.txt
- S3.1rbsareabyvariantlinkageall10.csv
- SIGuide.pdf
- S3.2af3rbsgeompoints.csv
- S1.3Phenotypediversityallseq.csv.csv
- rosettabenchmarkingstatsoverall.csv
- S1.2masterDNA13000phenotypes.csv
- S4.4top10variantmembersfinal.csv
- S2.1gantttable.csv
- S1.8top10variantgenoflucounts.csv
- S4.3mmgbsasummarystatsallcleaned.csv
- reference2021AAseq.txt
- S1.5PhenotypediversitylineageBseq.csv.csv
- S2.2hoststratifiedcoremetrics.csv
- S1.4PhenotypediversitylineageAseq.csv
- S4.2rbsregiondeltalong.csv
- S4.1rbsallresiduesdeltalong.csv
- rosettanumbering.csv
- S1.1masterAA2195phenotypes.csv
- Mubassiretal2026NATURESupplementaryInformation.pdf
- GISAIDacknowledgementtable.csv
- rosettabenchmarkingallpredictions.csv

## Data Availability

All Python scripts, processed datasets, and additional analysis outputs generated for this study are available at https://github.com/mhmmubassir/h5_2344b_USA. Genome sequences and associated metadata analyzed in this study were accessed through the GISAID EpiFlu database and are available through GISAID under EPI_SET identifier. All metadata underlying the analyses reported here are also provided in Supplementary Data. A complete acknowledgment table for all GISAID genomes used in this study is provided in the GitHub repository.
